# Prevention effect of quercetin and its glycosides on obesity and hyperglycemia through activating AMPKα in high-fat diet-fed ICR mice

**DOI:** 10.3164/jcbn.20-47

**Published:** 2020-06-05

**Authors:** Hao Jiang, Yuko Horiuchi, Ken-yu Hironao, Tomoya Kitakaze, Yoko Yamashita, Hitoshi Ashida

**Affiliations:** 1 Department of Agrobioscience, Graduate School of Agricultural Science, Kobe University, 1-1 Rokkodai-cho, Nada-ku, Kobe 657-8501, Japan

**Keywords:** quercetin, quercetin glycosides, glucose metabolism, lipid metabolism, AMP-activated protein kinase

## Abstract

Quercetin and its glycosides possess various health beneficial functions, but comparative study of them on energy metabolism in different tissues are not well studied. In this study, we investigated AMP-activated protein kinase regulated glucose metabolism in the skeletal muscle and lipid metabolism in the white adipose tissue and liver to compare the effectiveness of quercetin and its glycosides, namely isoquercitrin, rutin, and enzymatically modified isoquercitrin, in male ICR mice. The mice were fed a standard or high-fat diet supplemented with 0.1% quercetin and its glycosides for 13 weeks. Quercetin glycosides, but not quercetin, decreased body weight gain and fat accumulation in the mesenteric adipose tissue in high-fat groups. All compounds decreased high-fat diet-increased plasma glucose and insulin levels. Moreover, all compounds significantly increased AMP-activated protein kinase phosphorylation in either standard or high-fat diet-fed mice in all tissues tested. As its downstream events, all compounds induced glucose transporter 4 translocation in the muscle. In the white adipose tissue and liver, all compounds increased lipogenesis while decreased lipolysis. Moreover, all compounds increased browning markers and decreased differentiation markers in adipose tissue. Therefore, quercetin and its glycosides are promising food components for prevention of adiposity and hyperglycemia through modulating AMP-activated protein kinase-driven pathways.

## Introduction

The prevalence of obesity is increasing alarmingly over the past 25 years, which is closely related to the genetics and unhealthy lifestyles, such as low physical activity and excess consumption of lipids.^(1)^ Obesity is a heterogeneous disorder with a spectrum of traits and central to metabolic syndrome. The clinical manifestation of obesity is usually associated with insulin resistance accopanied by hyperglycemia, dyslipidemia including hypertriglyceridaemia, and subclinical inflammation in human.^(2–4)^ AMP-activated protein kinase (AMPK) modulates lipid accumulation and gene transcription, as well as the regulation of glucose metabolism to maintain the metabolic energy balance of the whole body.^(5)^ AMPK is ubiquitously expressed in various tissues and regulates various functions under the different conditions.^(6)^ AMPK not only regulates glucose metabolism and insulin sensitivity to maintain the glucose homeostasis in the muscle, but also regulates lipid metabolism including lipolysis, lipogenesis, fatty acid oxidation, and adipogenesis in the white adipose tissue (WAT) and liver.^(7)^ In skeletal muscles, AMPK facilitates glucose uptake through induces glucose transporter 4 (GLUT4) translocation by promoting phosphorylation of Rab-GTPase-activating protein TBC1D1, independent of the insulin-pathway.^(8)^ In WAT, AMPK regulates the expression level of carnitine palmitoyltransferase 1 (CPT1), acetyl-CoA carboxylase (ACC), sterol regulatory element binding protein 1 (SREBP1), peroxisome proliferator activated receptor (PPAR) γ, and CCAAT/enhancer binding proteins (C/EBPs).^(7)^ Uncoupling protein (UCP) 2 and CPT1, downstream factors of AMPK, are reported to be the regulators of mitochondrial fatty acid oxidation.^(7)^ SREBP1, PPARγ, and C/EBPs are associated with lipogenesis and adipocyte differentiation.^(7,9)^ UCP1,^(10)^ PPARγ coactivator-1α (PGC-1α),^(11)^ and PR-domain containing protein 16 (PRDM16),^(12)^ are involved in the regulation of mitochondrial biogenesis, thermogenesis and browning of WAT. In the liver, AMPK inhibits fatty acid synthesis, enhances fatty acid oxidation and improves lipid homeostasis through inhibition of ACC and activation of CPT1 and PPARα.^(13)^ In addition, hepatic glucose metabolism is also regulated by AMPK phosphorylation.^(14)^ Hence, the activation of AMPK would be an expectable event for reducing the risk of obesity and diabetes.

Quercetin, the most common polyphenol in edible plants, is presents as the various glycoside forms in natural food, such as quercetin-3-*O*-β-glucoside (isoquercitrin) and quercetin-3-*O*-β-rutinoside (rutin). Enzymatically modified isoquercitrin (EMIQ), derives from rutin via enzymatic hydrolysis, is a glycoside form of quercetin and has been recognized to be a safe food additive with high bioavailability.^(15)^ EMIQ is absorbed into the body similar to quercetin glycosides from natural sources.^(15)^ These quercetin glycosides possess various favorable effects against hyperglycemia, obesity and cancers.^(16–18)^ In recent years, epidemiological evidences and several clinical studies have demonstrated that quercetin shows the protective effect on DNA damage in alloxan induced type 2 diabetic mice;^(16)^ isoquercitrin inhibits adipocyte differentiation in 3T3-L1 cells;^(17)^ rutin inhibits proliferation and decreases migration of human cancerous cells:^(18)^ and EMIQ reveals anti-allergic effects in mice.^(19)^ Furthermore, it has been reported that quercetin glycosides received metabolic conversion to the glucuronide- and sulfate-conjugation forms, and only the slight amounts of quercetin aglycone exists in the body after long-term supplementation with quercetin glycosides.^(20)^ However, the underlying mechanisms of quercetin and its glycosides suppress hyperglycemia and obesity induced by high-fat (HF)-diet are not fully understood yet. In the present study, the effects of quercetin and its glycosides (isoquercitrin, rutin and EMIQ) on anti-hyperglycemia and anti-obesity in mice were compared and elucidated AMPK-driven mechanisms.

## Materials and Methods

### Materials

Quercetin, rutin, and commercial assay kits (Cholesterol-E test, Triglyceride-E test, NEFA-C test and Lab assay^TM^ Glucose Wako Kit) were purchased from Wako Pure Chemical Industries (Osaka, Japan). EMIQ and isoquercitrin were products of San-Ei Gen F. F. I. Co., Ltd. (Osaka, Japan). Protease and phosphatase inhibitor cocktails were produced by Roche Diagnostics (Tokyo, Japan). For Western blotting analysis, anti-ACC rabbit IgG, anti-phospho-ACC rabbit IgG, anti-AMPKα rabbit IgG, anti-phospho-AMPKα (Thr 172) rabbit IgG, anti-fatty acid synthase (FAS) rabbit IgG, anti-GLUT4 mouse IgG, anti-mouse IgG, and anti-rabbit IgG antibodies were obtained from Cell Signaling Technology (Denver, MA). Anti-β-actin rabbit IgG antibody was from Sigma Chemical (St. Louis, MO). Purified anti-UCP2 rabbit IgG was from BioLegend (San Diego, CA). Anti-PGC-1α mouse mAb, and anti-PRDM16 rabbit IgG, antibodies were from Millipore (Tokyo, Japan). Anti-C/EBPα (14AA) rabbit IgG, anti-C/EBPβ (C-19) rabbit IgG, anti-PPARα (H-98) rabbit IgG, anti-PPARγ (H-100) rabbit IgG, and anti-UCP1 (M-17) goat IgG antibodies were from Santa Cruz Biotechnology (Santa Cruz, CA). Anti-CPT1 mouse mAb and anti-SREBP1 rabbit IgG antibodies were from Abcam (Cambridge, MA). All other reagents used were of the highest grade available from commercial sources.

### Animal treatment

All animal experiments were approved by the Kobe University Institutional Animal Care and Use Committee (Permission #1-27-05-09) and carried out according to the guidelines for animal experiments at Kobe University Animal Experimentation Regulation. Male ICR mice (4 weeks old) were obtained from Japan SLC, Inc. (Shizuoka, Japan) and maintained in an air-conditioned room (23 ± 2°C) under a 12 h light-dark cycle with free access to water and commercial chow (Research Diets, Tokyo, Japan) for 7 days. Mice were randomly divided into ten groups and fed a standard or HF diet containing 30% (w/w) lard with quercetin, isoquercitrin, rutin, and EMIQ at 0 or 0.1% (w/w) for 13 weeks. The standard and HF diet fed groups were refed as C-0 and HF-0, respectively. Body weight was monitored every week. At the end of the experiment, mice were sacrificed and plasma, liver, skeletal muscle (soleus and gastrocnemius), white adipose tissue (WAT) and brown adipose tissue (BAT) were collected. The tissues and plasma were frozen in liquid N_2_ and stored at −80°C until analyzed.

### Measurement of plasma parameters

Plasma triacylglycerol, total cholesterol, non-esterified fatty acid (NEFA) and glucose levels were measured using the corresponding commercial kit. Plasma insulin was also measured using the commercial ELISA kit (Mouse Insulin ELISA Kit, Shibayagi, Gunma, Japan). Insulin resistance index (HOMA-IR) was calculated using the following formula: HOMA-IR = fasting plasma glucose (mg/dl) × fasting plasma insulin (ng/ml)/405.^(21)^

### Preparation of the plasma membrane fraction and tissue lysate

For Western blotting, the plasma membrane fraction and tissue lysate from WAT, liver, and muscle were prepared for the measurement of related signal pathways as described in the previous study.^(22)^

### Western blotting

Equal amounts of proteins in tissue lysate and plasma membrane fraction were separated by sodium dodecyl sulfate polyacrylamide gel electrophoresis as described previously.^(23)^ ImmunoStar LD Chemiluminescence Detection kit and Light-Capture II (ATTO Corp, Tokyo, Japan) were used to visualize the protein bands. Then, ImageJ software (NIH, Bethesda, MD) was used to determine the density of specific protein band.

### Statistical analysis

Data are represented as the means and SE. The statistical analysis was performed using Tukey-Kramer multiple comparison test using JMP 11.2.0 (SAS, Cary, NC). *P*<0.05 was considered statistically significant.

## Results

### Effects of quercetin and its glycosides on body and tissue weight of mice

The preventive effects of quercetin, and its glycosides (isoquercitrin, rutin, and EMIQ) on hyperglycemia and obesity were investigated after 13 weeks feeding. As shown in Table 1, body weight of the mice significantly increased in the HF-0 group as expected. This body weight gain was suppressed by supplementation with quercetin glycosides but not with quercetin. Supplementation with isoquercitrin was the most effective on the decrease of body weight in HF-diet fed group. An intake of HF-diet significantly increased in the weight of all WATs (epididymal, mesenteric, retroperitoneal, subcutaneous adipose tissue) compared with the intake of standard diet. Quercetin glycosides significantly decreased the weight of mesenteric WAT. Quercetin and its glycosides tended to decrease the weight of the other WATs. These results indicated that long-term feeding of quercetin glycosides have the potential to reduce HF diet-induced body weight and fat accumulation in WATs.

### The effects of quercetin and its glycosides on the plasma glucose and lipid levels

**Fig. 1:**
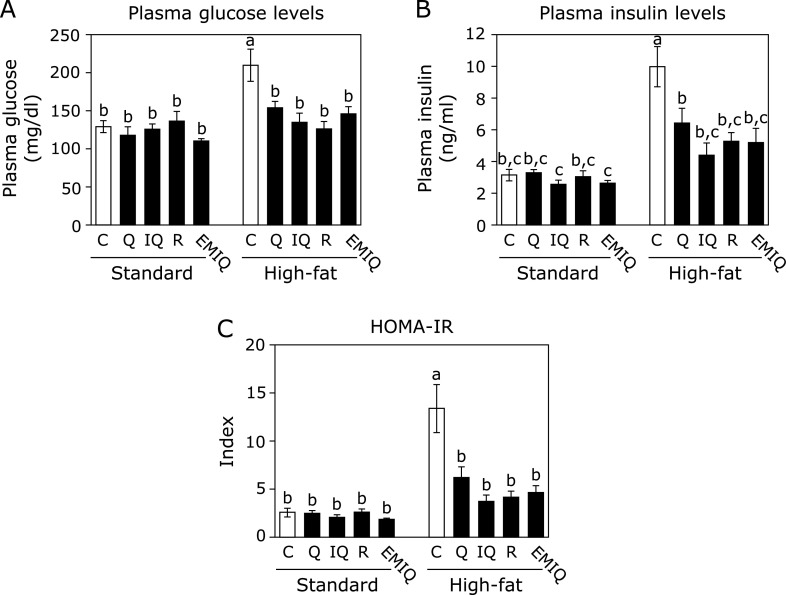
The effects of quercetin (Q), isoquercitrin (IQ), rutin (R), and EMIQ on the plasma glucose, and insulin levels. The levels of (A) plasma glucose and (B) insulin were measured and (C) the HOMA-IR was calculated. Values were shown as the mean ± SE (*n* = 5). Different letters indicate significant differences among the groups by Tukey-Kramer multiple comparison test (*p*<0.05).

After 13 weeks feeding, the plasma glucose and insulin levels in the HF-0 group were significantly higher than those in the C-0 group, indicating that hyperglycemia and hyperinsulinemia were induced by HF diet as expected (Fig. 1A). Supplementation with quercetin and its glycosides drastically reduced HF diet-induced hyperglycemia and hyperinsulinemia to the level of C-0 group (Fig. 1A and B). The HOMA-IR index in the HF-0 group was significantly higher than that in the C-0 group. Quercetin and its glycosides significantly decreased HOMA-IR index in the HF diet-fed groups (Fig. 1C). The total plasma cholesterol level was also significantly higher in the HF-0 group than in the C-0 group (Table 2). Supplementation with isoquercitrin, rutin and EMIQ significantly reduced HF diet-increased plasma cholesterol level, although quercetin did not show a significant change. These results indicated that not only quercetin glycosides, but also quercetin have the potential to prevent insulin resistance and quercetin glycosides also have the potential to improve hypercholesterolemia.

### Quercetin and its glycosides promote AMPK phosphorylation in the skeletal muscle, adipose tissue, and liver

**Fig. 2:**
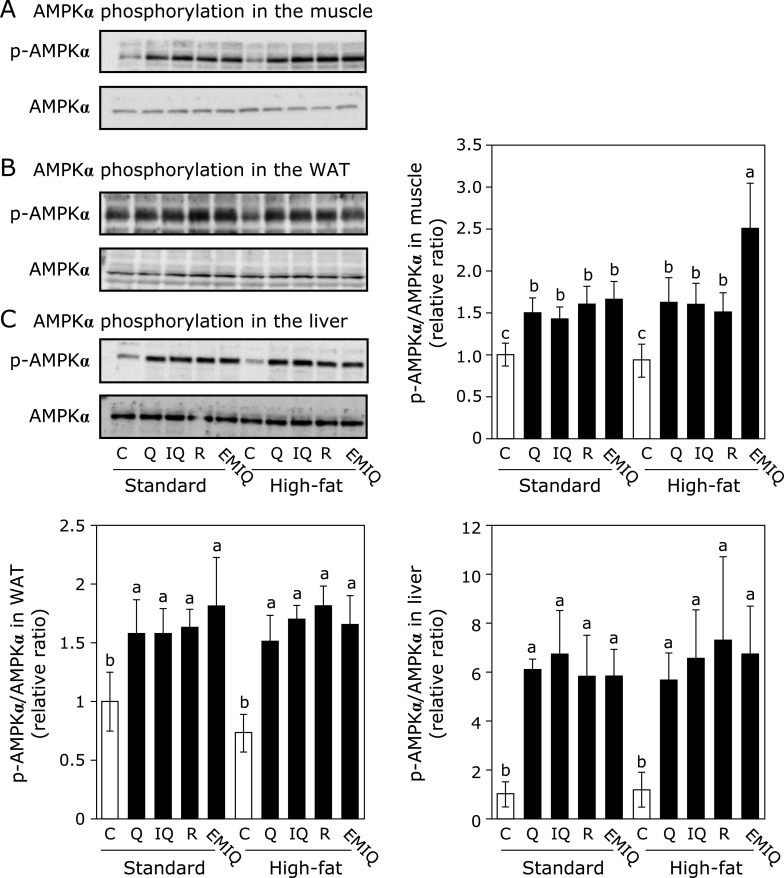
Effect of quercetin (Q), isoquercitrin (IQ), rutin (R), and EMIQ on AMPK phosphorylation in the muscle, WAT, and liver. The phosphorylation and expression of AMPK in tissue lysates of (A) skeletal muscle, (B) WAT, and (C) liver were determined by Western blot analysis. Each panel shows a typical blot from five animals. The normalized density of specific protein band shown in the bar graphs. Values were shown as the mean ± SE (*n* = 5). Different letters indicate significant differences among the groups by Tukey-Kramer multiple comparison test (*p*<0.05).

AMPK plays a pivotal role in regulating energy metabolism including glucose metabolism.^(5,24)^ Furthermore, it is reported that the intake of HF diet suppressed the AMPK activation in WAT.^(25)^ Therefore, we investigated whether the supplementation with quercetin and its glycosides affected AMPK phosphorylation to maintain glucose and lipid homeostasis. Supplementation with quercetin and its glycosides increased phosphorylation of AMPK without affecting its expression level in the skeletal muscle, WAT, and liver (Fig. 2). From this result, it is suggested that quercetin and its glycosides improved HF diet-caused hyperglycemia and fat accumulation through promoting phosphorylation of AMPK in these tissues.

### Quercetin and its glycosides induced GLUT4 translocation in skeletal muscle

**Fig. 3:**
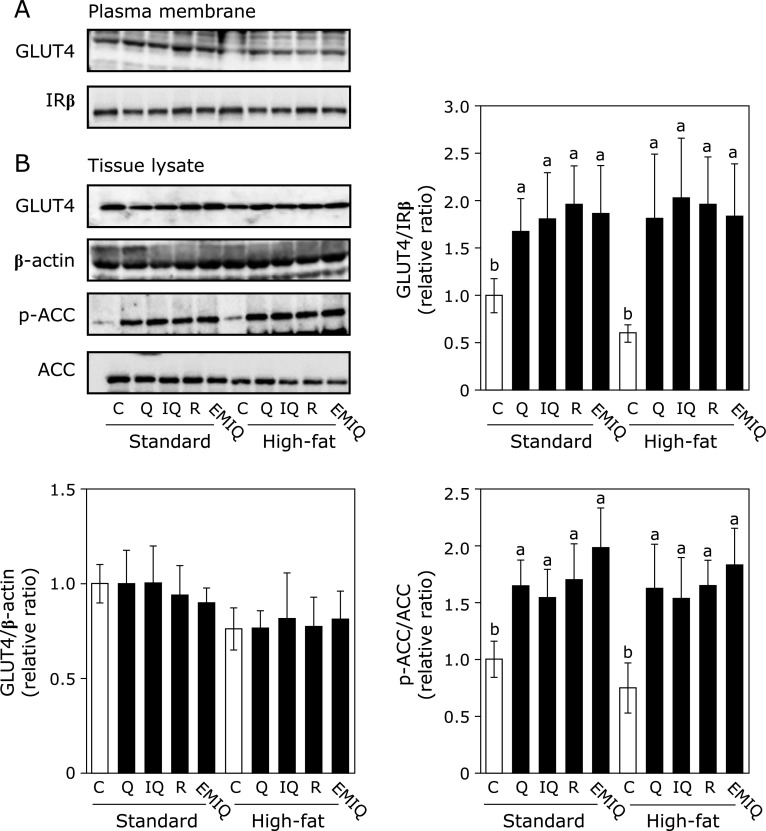
Effect of quercetin (Q), isoquercitrin (IQ), rutin (R), and EMIQ on the translocation of GLUT4 in skeletal muscle. GLUT4, IR-β, β-actin, phosphorylated-ACC and ACC protein expression in (A) the plasma membrane fraction and (B) tissue lysate of skeletal muscle were determined by Western blot analysis. Each panel shows a typical blot from five animals. The normalized density of specific protein band shown in the bar graphs. Values were shown as the mean ± SE (*n* = 5). Different letters indicate significant differences among the groups by Tukey-Kramer multiple comparison test (*p*<0.05).

As a downstream event of AMPK phosphorylation, GLUT4 translocation in the plasma membrane was investigated in skeletal muscle to understand the prevention mechanism of quercetin and its glycosides against insulin resistance and hyperglycemia. As shown in Fig. 3, quercetin and its glycosides significantly increased GLUT4 translocation to the plasma membrane of skeletal muscle in both standard and HF diet groups (Fig. 3). It was observed that all compounds also promoted phosphorylation of ACC as another downstream factor of AMPK phosphorylation. In addition to AMPK-dependent pathway, insulin- and JAK/STAT-pathways are involved in GLUT4 translocation.^(26,27)^ However, all compounds did not affect these pathways (Data not shown). These results indicated that quercetin and its glycosides promoted GLUT4 translocation to the plasma membrane through AMPK phosphorylation, resulting in normalization of the blood glucose level.

### Quercetin and its glycosides suppressed adiposity-related metabolism in the adipose tissue

**Fig. 4:**
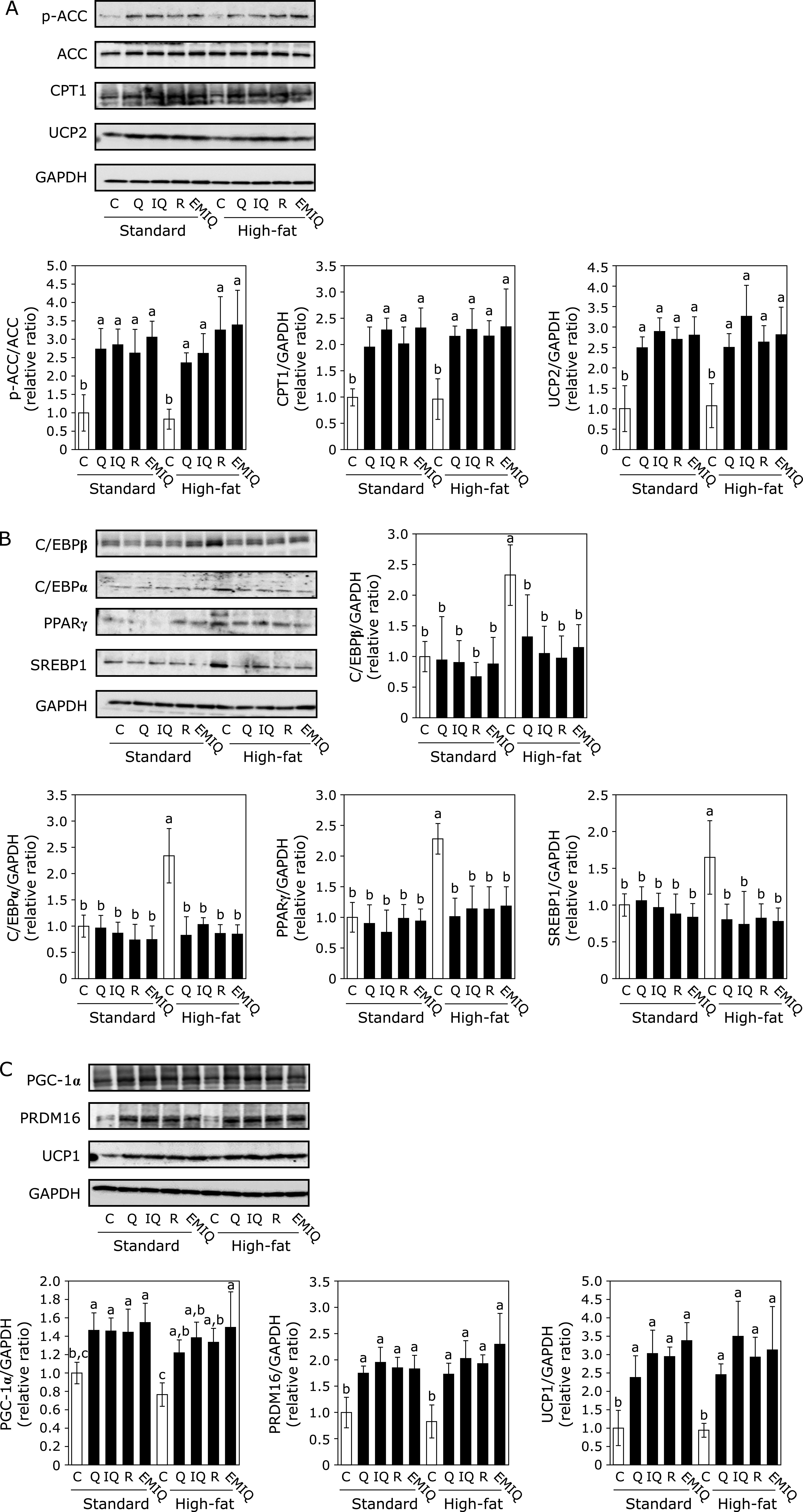
Effect of quercetin (Q), isoquercitrin (IQ), rutin (R), and EMIQ on the expression of adiposity-related proteins in WAT. The expression of (A) p-ACC, ACC, CPT1, UCP2, and GAPDH in the tissue lysate of WAT, the expression of (B) C/EBPβ, C/EBPα, PPARγ, SREBP1 and GAPDH in tissue lysate of WAT and the expression of (C) PGC-1α, PRDM16, UCP1,and GAPDH in tissue lysate of WAT were determined by Western blot analysis. Each panel shows a typical blot from five animals. The normalized density of specific protein band shown in the bar graphs. Values were shown as the mean ± SE (*n* = 5). Different letters indicate significant differences among the groups by Tukey-Kramer multiple comparison test (*p*<0.05).

Next, we investigated the mechanism of quercetin and its glycosides on adiposity-related metabolism in the WAT. As shown in Fig. 4A, quercetin and its glycosides supplementation increased ACC phosphorylation in the WAT, concomitant with AMPK phosphorylation in both standard- and HF-diet groups. The expression level of UCP2 and CPT1 were significantly increased in the quercetin and its glycosides groups compared with the C-0 and HF-0 groups (Fig. 4A). These results indicate that quercetin and its glycosides suppress lipogenesis while promoted lipolysis under the regulation of AMPK.

As to the adipocyte differentiation markers, the expression level of C/EBPβ, C/EBPα, PPARγ and SREBP1 were significantly increased in the HF-0 group compared with the standard diet groups (Fig. 4B). In the HF diet groups, supplementation with quercetin and its glycosides significantly suppressed the increased expression of these proteins in HF groups. These results indicate that quercetin and its glycosides suppress adipocyte differentiation through AMPK phosphorylation.

UCP1, PGC-1α and PRDM16 are reported to be associated with lipid metabolism, thermogenesis and browning.^(28–30)^ Therefore, the expression level of these protein in WAT were also determined. As shown in Fig. 4C, supplementation with quercetin and its glycosides significantly increase the expression levels of these proteins in WAT in both standard- and HF-diet groups compared with the C-0 and HF-0 groups. From these results, quercetin and its glycosides have the ability to suppress HF diet-induced fat accumulation by increasing energy expenditure due to browning of WAT through promoting AMPK phosphorylation.

### Quercetin and its glycosides modulated lipid metabolism in the liver

**Fig. 5:**
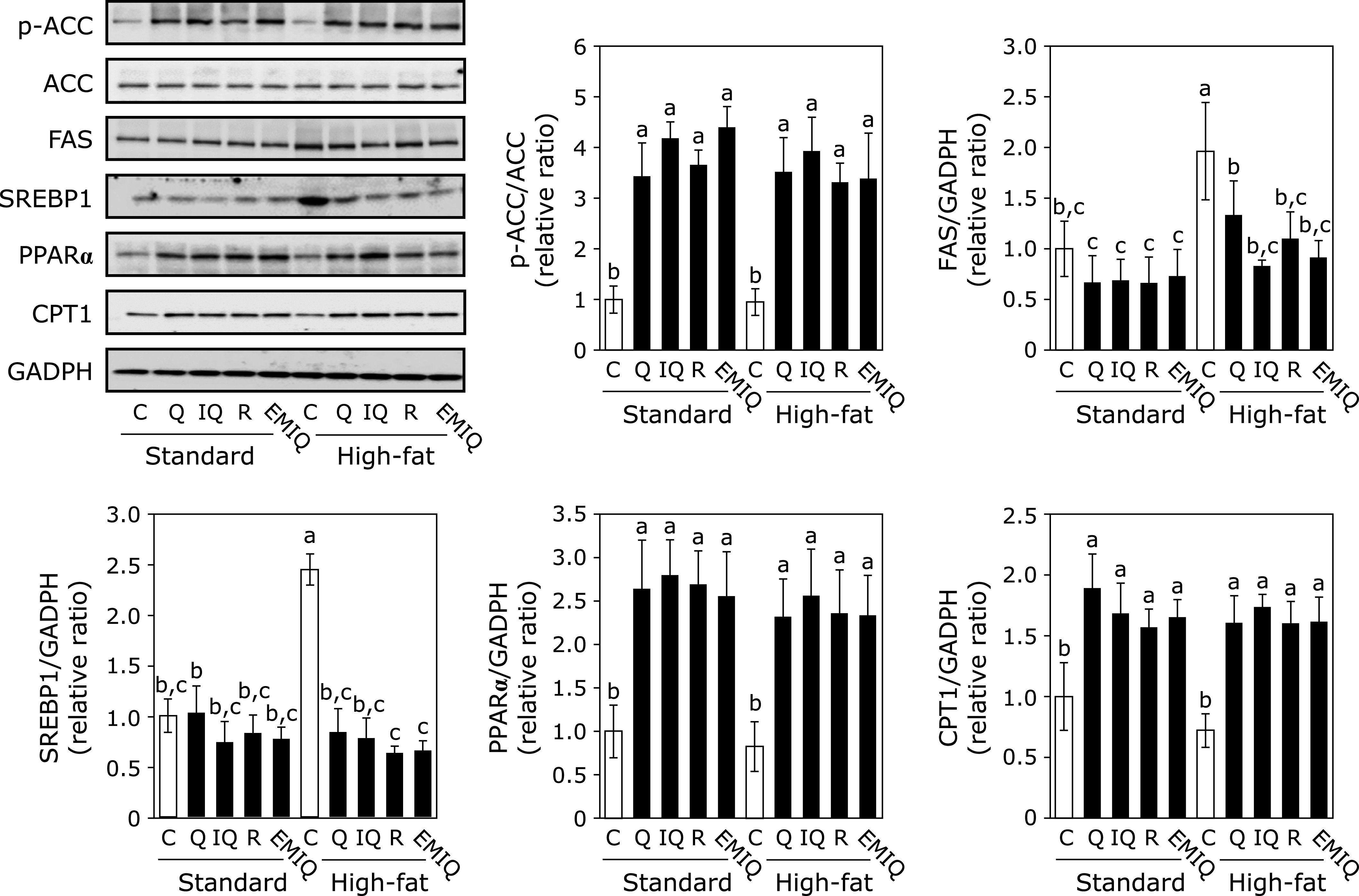
Effect of quercetin (Q), isoquercitrin (IQ), rutin (R), and EMIQ on the expression of lipid metabolism-related proteins in the liver. The expression of p-ACC, ACC, FAS, SREBP1, PPARα, CPT1 and GAPDH in tissue lysate of liver were determined by Western blot analysis. Each panel shows a typical blot from five animals. The normalized density of specific protein band shown in the bar graphs. Values were shown as the mean ± SE (*n* = 5). Different letters indicate significant differences among the groups by Tukey-Kramer multiple comparison test (*p*<0.05).

Since the liver is also contributed to lipid metabolism, it was investigated that the expression level of FAS, SREBP1, PPARα and CPT-1, in addition to the phosphorylation level of ACC in the liver. As shown in Fig. 5, we confirmed phosphorylation level of ACC was increased by quercetin and its glycosides in both standard- and HF-diet groups. FAS and SREBP1 were increased by the HF diet and supplementation with quercetin and its glycosides significantly decreased their increased expression. Supplementation of quercetin and its glycosides significantly increased the expression level of CPT1 and PPARα in the liver in both standard- and HF-diet groups. These results indicate that quercetin and its glycosides also modulate lipid metabolism in the liver, in addition to in the WAT.

## Discussion

Excessive energy intake causes obesity through disrupting energy metabolism. Obesity is recognized as a risk factor for the development of various diseases including T2DM.^(2,31)^ The pathophysiology of T2DM is chronic hyperglycemia, which causes a deterioration in insulin sensitivity.^(2,3)^ Thus, prevention of obesity and hyperglycemia by food factors is important to maintain human health. In this study, we demonstrated that the intake of quercetin and its glycosides for long-term prevented chronic hyperglycemia and obesity in the mice through AMPK-driven pathways.

In the present study, supplementation with both 0.1% quercetin and its glycosides for 13 weeks promoted AMPK phosphorylation in the skeletal muscle, WAT, and liver (Fig. 2). On the other hand, our previous study demonstrated that 0.02 and 0.1% EMIQ, one of the quercetin glycosides, for 2 weeks also promoted AMPK phosphorylation in the skeletal muscle, WAT, and liver, but quercetin did not promote AMPK phosphorylation.^(24)^ This discrepancy was due to difference in the effective concentration of quercetin aglycone in these tissues as one of the possible explanations. It is known that the bioavailability of quercetin is poor, while that of the glycosides are relatively higher than the aglycone.^(15)^ Indeed, our previous results demonstrated that quercetin aglycone did not appear in the plasma after administration of quercetin itself, but the concentration of quercetin aglycone form was 6.8 nM in the plasma and 0.26 nmol/g in the muscle 90 min after a single administration of EMIQ at 100 mg/kg body weight.^(27)^ However, after supplementation with 0.1% quercetin for 11 weeks in the rats, quercetin aglycone and its metabolite isorhamnetin were detected in the plasma, liver and skeletal muscle.^(32)^ These data indicated that a slight amount of quercetin aglycone is detected in the body after long-term feeding. Another possible explanation is the internal metabolic conversion from the conjugation forms to aglycone in the body. It was reported that β-glucuronidase can convert the conjugated forms of quercetin to aglycone under certain physiologic conditions, such as inflammation and neoplasm.^(11,12,15)^ It was also reported that quercetin 3-*O*-β-glucuronide and quercetin 7-*O*-β-glucuronide were deconjugated and further metabolized to quercetin 3'-*O*-sulfate and quercetin aglycone by β-glucuronidase in hepatocytes.^(33)^ Furthermore, obesity is associated with inflammation.^(1)^ Therefore, the deconjugation reaction may occurred in the mice, in particular high-fat diet given mice in this study.

It is known that quercetin and its glycosides prevented hyperglycemia. For example, dietary supplementation with quercetin prevented HF diet-induced hyperglycemia after 8-weeks feeding.^(34)^ Isoquercitrin ameliorated hyperglycemia and regulated the key enzymes for glucose metabolism in diabetic rats.^(35)^ Similarly, the anti-hyperglycemic effect was also observed after oral administration of rutin (50 mg/kg body weight).^(36)^ As to the mechanism for anti-hyperglycemia in this study, GLUT4 translocation in the skeletal muscle was involved in the downstream event for AMPK phosphorylation (Fig. 3) without activating insulin- and JAK/STAT-pathways (data not shown). This result is consistent with our previous report that EMIQ promotes GLUT4 translocation by the AMPK pathway, but not by insulin- and JAK/STAT-pathways.^(24)^ It is reported that AMPK is activated by phosphorylation of its threonine residue (Thr172) by the contraction of skeletal muscle and the reduction in the ratios of ATP/AMP and creatine/phosphocreatine.^(37)^ Furthermore, liver kinase B1, calcium/calmodulin-dependent protein kinase kinase II, and TGFβ-activated kinase 1 are the upstream factors of AMPK phosphorylation.^(27,38)^ Hence, the upstream event for quercetin- and its glycosides-induced AMPK phosphorylation should be explored in future.

Fat accumulation and lipid metabolism are tightly controlled by the adipose tissue and liver.^(39,40)^ AMPK is a key molecule to maintain energy balance. Upon the activation, AMPK suppresses adipocyte differentiation and lipogenesis and activates fatty acid oxidation and lipolysis through the regulation of the downstream targets.^(41)^ In the present study, we demonstrated that quercetin and its glycosides decreased lipogenesis and increased fatty acid oxidation and lipolysis under regulation of AMPK in the WAT and liver (Fig. 4 and 5). ACC is a well-known target for AMPK and phosphorylation of ACC leads to inhibit its activity and decrease the content of malonyl-CoA, resulting an increase in the CPT-1 activity through cancelling the inhibitory effect of malonyl-CoA against CPT-1.^(42,43)^ In addition, quercetin and its glycosides suppressed HF diet-increased SREBP1 and FAS expression (Fig. 4 and 5). SREBP1 is a key lipogenic transcription factor to regulate *de novo* lipogenesis.^(23)^ SREBP1 also cooperates with FAS to modulate hepatic fatty acid and triglyceride synthesis.^(44)^ ACC, CPT1 and PPARα are also involved in fatty acid oxidation.^(7)^ Meanwhile, lipid accumulation was downregulated by the PPARα agonist in the liver of rats.^(45)^ The data are similar to the previous results that ashitaba extract inhibited the lipid accumulation through downregulating SREBP1 and upregulating PPARα.^(23)^ These results indicate that quercetin and its glycosides regulate lipid metabolism in the WAT and liver through increasing fatty acid oxidation and lipolysis and decreasing lipogenesis.

We also found quercetin and its glycosides suppressed adipocyte differentiation (Fig. 4B). C/EBPs are critical transcription factors of lipogenesis and morphological modifications.^(7)^ C/EBPβ is the first transcription factor to be involved in directing the differentiation process: The transcription and expression of C/EBPβ is increased in preadipocytes after treatment with the inducers for differentiation.^(46)^ C/EBPα is not only involved in adipogenesis of mature adipocytes, but also solidified the correlative link to adipose-specific genes, such as GLUT4, SCD1, leptin, and 422/aP2.^(47)^ It has been noted that C/EBPβ causes preadipocytes differentiation without increasing C/EBPα expression in pluripotent NIH 3T3 cells, indicating that C/EBPβ may functionally replace C/EBPα.^(48)^ In addition, multiple post-translational modifications have been reported to regulate C/EBPβ, including phosphorylation, acetylation, ubiquitination and sumoylation.^(49–51)^ Theobromine has been reported to induce C/EBPβ degradation by increasing its sumoylation at Lys133 in mice.^(52)^ Quercetin treatment increases SUMO-conjugation (both SUMO-1 and SUMO-2) in SHSY5Y cells and E18 rat cortical neurons.^(53)^ Further study is needed to clarify whether quercetin and its glycosides induced C/EBPβ degradation through its sumoylation.

Furthermore, supplementation with quercetin and its glycosides upregulated the browning makers in WAT (Fig. 4C). It has been reported that WAT can convert to BAT-like adipose tissue by a process called “browning or beiging” in response to prolonged cold exposure or β-adrenergic stimulation.^(54)^ During the aggravation of obesity, generation of beige adipocytes gradually decreased, which contributes to a decrease in energy expenditure, weaken the thermogenic capacity, and impair the insulin sensitivity.^(55)^ Hence, browning of WAT is a potential approach for anti-obesity therapy through regulating AMPK targets, including PGC-1α, PRDM16, and UCP1.^(11,12,29)^ In our previous study, supplementation with EMIQ increased the expression level of PGC-1α, PRDM16, and UCP1 through AMPK phosphorylation,^(24)^ which consistent with the results. In addition, Choi H also reported the similar results that quercetin upregulates UCP1, implying increased WAT browning and BAT activity, via the activation of the AMPK/PPARγ pathway *in vivo* and *in vitro*.^(56)^ Based on these results, browning of WAT is also involved in the prevention of adiposity by quercetin and its glycosides.

In this study, different effects between quercetin and its glycosides were observed: Quercetin did not decrease the HF diet-increased body weight gain, fat accumulation in mesenteric white adipose tissue, and the plasma cholesterol level (Table 1 and 2). These results suggest that quercetin was more effective for suppressing hyperglycemia than regulating body weight gain and fat accumulation. Previous reports demonstrated that consumption of quercetin at 2 mg/kg body weight attenuated HF diet-induced hyperglycemia, rather than affected the body weight in obese Zucker rats for 10 weeks.^(57)^ Contrarily, supplementation with 0.05% quercetin decreased HF diet-induced body weight gain after 20 weeks feeding.^(58)^ Thus, the preventive actions of quercetin against hyperglycemia and obesity is still controversial. Different effects quercetin and its glycosides may also due to the different bioavailability of these compounds. In this study, only mesenteric adipose tissue significantly decreased after 13 weeks feeding, though epididymal, retroperitoneal, and subcutaneous adipose tissues showed the decreasing tendency (Table 1). The reason is that mesenteric is visceral adipose tissue, which is more sensitive to weight reduction than the other adipose tissues.^(59)^ Similar result was reported that green tea extract (400 mg/kg body weight/day) significantly lowered the HF diet-increased the body weight gain in the mice accompanied by suppression of fat accumulation in the mesenteric adipose tissue.^(60)^ After intake of quercetin and its glycosides, quercetin glucuronides appeared mainly in mesenteric blood *in situ* intestinal perfusion of rats.^(61)^ These results illustrated that the possible reason why mesenteric adipose tissue weight was significantly decreased, rather than retroperitoneal, epididymal or subcutaneous adipose tissues.

In conclusion, our findings indicated that quercetin and its glycosides prevented HF diet-induced insulin resistance by promoting GLUT4 translocation in skeletal muscle, and also prevented obesity by activating AMPK-dependent signaling pathways in adipose tissue and liver. Therefore, quercetin and its glycosides are promising food components in the treatment of insulin resistance and obesity.

## Author Contributions

YY and HA conceived and designed the research; HJ, YH, and KH performed the experiments; HJ, TK, and HA analysed the data and wrote the manuscript.

## Figures and Tables

**Table 1:** Effect of quercetin, isoquercitrin, rutin and EMIQ on the body and tissue weight

	Standard diet						High-fat diet				
	Control	Quercetin	Isoquercitrin	Rutin	EMIQ		Control	Quercetin	Isoquercitrin	Rutin	EMIQ
Final body weight (g)	32.3 ± 1.4^b,c^	32.2 ± 0.4^b,c^	31.6 ± 0.8^c^	33.2 ± 1.6^b,c^	31 ± 0.6^c^		41 ± 2.3^a^	36.9 ± 0.8^a,b^	32.1 ± 1.3^b,c^	32.8 ± 2.6^b,c^	35.6 ± 2.6^b,c^
Lean weight (g)	19.8 ± 1.5^a^	19.1 ± 1.0^a^	19.4 ± 0.7^a^	20.2 ± 1.1^a^	18.9 ± 1.1^a^		19.3 ± 1.8^a^	17 ± 1.0^a^	15.3 ± 0.3^a^	16.7 ± 1.7^a^	16.2 ± 0.7^a^
Tissue weight (% of body weight)											
Liver	4.7 ± 0.3^a^	4.2 ± 0.7^a^	4.4 ± 0.3^a^	4.7 ± 0.3^a^	4.6 ± 0.3^a^		4.6 ± 0.5^a^	3.9 ± 0.2^a^	4.1 ± 0.3^a^	4.1 ± 0.2^a^	4 ± 0.5^a^
Adipose tissue weight											
Mesenteric	1.63 ± 0.25^b^	1.67 ± 0.07^b^	1.74 ± 0.08^b^	1.57 ± 0.16^b^	1.57 ± 0.09^b^		2.59 ± 0.36^a^	1.92 ± 0.07^a,b^	1.49 ± 0.08^b^	1.37 ± 0.14^b^	1.77 ± 0.12^b^
Retroperitoneal	1.53 ± 0.21^c^	1.7 ± 0.13^b,c^	1.71^c^ ± 0.11^b^	1.55 ± 0.14^b,c^	1.56 ± 0.17^b,c^		2.71 ± 0.17^a^	2.68 ± 0.15^a^	2.6 ± 0.16^a^	2.29 ± 0.11^a,b^	2.66 ± 0.13^a^
Epididymal	3.71 ± 0.55^c^	4.01 ± 0.39^b,c^	4 ± 0.18^b,c^	3.53 ± 0.20^c^	3.51 ± 0.42^c^		6.67 ± 0.23^a^	6.53 ± 0.43^a^	5.69 ± 0.47^a,b^	5.54 ± 0.29^a,b^	5.77 ± 0.27^a,b^
Subcutaneous	5.42 ± 0.71^c^	6.33 ± 0.44^b,c^	5.66 ± 0.41^c^	7.2 ± 0.49^a,b,c^	5.66 ± 0.61^c^		8.81 ± 0.53^a,b^	9.19 ± 0.69^a^	8.63 ± 0.62^a,b^	8.61 ± 0.84^a,b^	8.48 ± 0.64^a,b,c^
Brown	0.45 ± 0.07^a^	0.83 ± 0.12^a^	0.82 ± 0.10^a^	0.71 ± 0.09^a^	0.87 ± 0.14^a^		0.67 ± 0.11^a^	0.51 ± 0.08^a^	0.48 ± 0.03^a^	0.63 ± 0.11^a^	0.72 ± 0.06^a^

Mice were fed a standard or high-fat diet containing 0.1% quercetin and its glycosides for 13 weeks. At the end of the experiment, body, adipose tissue and liver weights were measured after an 18 h fasting. Values are the mean ± SE (*n* = 5). Values without a common letter in each row differ significantly among the groups (*p*<0.05) by Tukey-Kramer multiple comparison test.

**Table 2:** Effect of quercetin, isoquercitrin, rutin and EMIQ on the plasma lipid level

	Standard diet						High-fat diet				
	Control	Quercetin	Isoquercitrin	Rutin	EMIQ		Control	Quercetin	Isoquercitrin	Rutin	EMIQ
Cholesterol (mg/dl)	84.4 ± 4.5^c^	82.6 ± 3.8^c^	83.5 ± 3.1^c^	85.7 ± 6.6^c^	91.3 ± 4.4^c^		142.4 ± 12.9^a^	119.3 ± 6.6^a,b^	105.5 ± 3.5^b,c^	99.2 ± 4.4^b,c^	107.8 ± 6.0^b,c^
Triglyceride (mg/dl)	130.7 ± 20.1^a^	139.7 ± 17.1^a^	126.7 ± 16.8^a^	114.2 ± 17.3^a^	139.9 ± 15.3^a^		128.1 ± 15.8^a^	137 ± 13.7^a^	141.1 ± 12.1^a^	121.2 ± 5.7^a^	124 ± 13.9^a^
NEFA (mEq/L)	0.76 ± 0.10^a^	0.95 ± 0.13^a^	0.8 ± 0.09^a^	0.79 ± 0.08^a^	0.89 ± 0.04^a^		0.66 ± 0.05^a^	0.79 ± 0.06^a^	0.69 ± 0.05^a^	0.74 ± 0.04^a^	0.76 ± 0.08^a^

Mice were fed a standard or high-fat diet containing 0.1% quercetin and its glycosides for 13 weeks. At the end of the experiment, plasma levels of total cholesterol, triglyceride and NEFA were measured after an 18 h fasting. Values are the mean ± SE (*n* = 5). Values without a common letter in each row differ significantly among the groups (*p*<0.05) by Tukey-Kramer multiple comparison test.

## Abbreviations

ACC: acetyl-CoA carboxylase
AMPK: adenosine monophosphate-activated protein kinase
BAT: brown adipose tissue
C/EBP: CCAAT/enhancer-binding protein
CPT1: carnitine palmitoyltransferase 1
FAS: fatty acid synthase
GLUT4: glucose transporter 4
HF: high fat
HOMA-IR: homeostasis model assessment of insulin resistance
JAK: Janus kinase
PGC-1α: peroxisome proliferator-activated receptor gamma coactivator-1alpha
PPAR: peroxisome proliferator-activated receptor
PRDM16: PR domain containing 16
SREBP: sterol regulatory element-binding protein
STAT: signal transducer and activator of transcription
UCP: uncoupling protein
WAT: white adipose tissue

## References

[1] Bhurosy T, Jeewon R Overweight and obesity epidemic in developing countries: a problem with diet, physical activity, or socioeconomic status? ScientificWorldJournal 2014; 2014: 964236. 25379554 10.1155/2014/964236 PMC4212551

[2] Tuomilehto J, Lindström J, Eriksson JG, et al.; Finnish Diabetes Prevention Study Group Prevention of type 2 diabetes mellitus by changes in lifestyle among subjects with impaired glucose tolerance. N Engl J Med 2001; 344: 1343–1350. 11333990 10.1056/NEJM200105033441801

[3] Bruun JM, Lihn AS, Pedersen SB, Richelsen B Monocyte chemoattractant protein-1 release is higher in visceral than subcutaneouos human adipose tissue (AT): implication of macrophages resident in the AT. J Clin Endocrinol Metab 2005; 90: 2282–2289. 15671098 10.1210/jc.2004-1696

[4] Havel PJ Update on adipocyte hormones: regulation of energy balance and carbohydrate/lipid metabolism. Diabetes 2004; 53 Suppl 1: S143–S151. 14749280 10.2337/diabetes.53.2007.s143

[5] Long YC, Zierath JR AMP-activated protein kinase signaling in metabolic regulation. J Clin Invest 2006; 116: 1776–1783. 16823475 10.1172/JCI29044 PMC1483147

[6] Dasgupta B, Chhipa RR Evolving lessons on the complex role of AMPK in normal physiology and cancer. Trends Pharmacol Sci 2016; 37: 192–206. 26711141 10.1016/j.tips.2015.11.007 PMC4764394

[7] Cho BY Park MR Lee JH Standardized cirsium setidens nakai ethanolic extract suppresses adipogenesis and regulates lipid metabolisms in 3T3-L1 adipocytes and C57BL/6J mice fed high-fat diets. J Med Food 2017; 20: 763–776. 28686516 10.1089/jmf.2017.3965

[8] Chavez JA, Roach WG, Keller SR, Lane WS, Lienhard GE Inhibition of GLUT 4 translocation by TBC1D1, a Rab GTPase-activating protein abundant in skeletal muscle, is partially relieved by AMP-activated protein kinase activation. J Biol Chem 2008; 283: 9187–9195. 18258599 10.1074/jbc.M708934200 PMC2431020

[9] Moseti D, Regassa A, Kim WK Molecular regulation of adipogenesis and potential anti-adipogenic bioactive molecules. Int J Mol Sci 2016; 17. pii: E124. 26797605 10.3390/ijms17010124 PMC4730365

[10] Cryer PE Axelrod L Grossman AB Evaluation and management of adult hypoglycemic disorders: an Endocrine Society Clinical Practice Guideline. J Clin Endocrinol Metab 2009; 94: 709–728. 19088155 10.1210/jc.2008-1410

[11] Shimoi K, Nakayama T Glucuronidase deconjugation in inflammation. Method Enzymol 2005; 400: 263–272. 10.1016/S0076-6879(05)00015-7 16399354

[12] Yuan L Wagatsuma C Yoshida M Inhibition of human breast cancer growth by GCP (genistein combined polysaccharide) in xenogeneic athymic mice: involvement of genistein biotransformation by beta-glucuronidase from tumor tissues. Mutat Res 2003; 523–524: 55–62. 10.1016/s0027-5107(02)00321-4 12628503

[13] Viollet B Foretz M Guigas B Activation of AMP-activated protein kinase in the liver: a new strategy for the management of metabolic hepatic disorders. J Physiol 2006; 574 (Pt 1): 41–53. 16644802 10.1113/jphysiol.2006.108506 PMC1817784

[14] Miao J, Fang S, Bae Y, Kemper JK Functional inhibitory cross-talk between constitutive androstane receptor and hepatic nuclear factor-4 in hepatic lipid/glucose metabolism is mediated by competition for binding to the DR1 motif and to the common coactivators, GRIP-1 and PGC-1α. J Biol Chem 2006; 281: 14537–14546. 16492670 10.1074/jbc.M510713200

[15] Murota K Matsuda N Kashino Y α-Oligoglucosylation of a sugar moiety enhances the bioabailability of quercetin glucosides in humans. Arch Biochem Biophys 2010; 501: 91–97. 20638359 10.1016/j.abb.2010.06.036

[16] Alam MM, Meerza D, Naseem I Protective effect of quercetin on hyperglycemia, oxidative stress and DNA damage in alloxan induced type 2 diabetic mice. Life Sci 2014; 109: 8–14. 24946265 10.1016/j.lfs.2014.06.005

[17] Lee SH Kim B Oh MJ *Persicaria hydropiper* (L.) spach and its flavonoid components, isoquercitrin and isorhamnetin, activate the Wnt/β-catenin pathway and inhibit adipocyte differentiation of 3T3-L1 cells. Phytother Res 2011; 25: 1629–1635. 21413092 10.1002/ptr.3469

[18] Ben Sghaier M, Pagano A, Mousslim M, Ammari Y, Kovacic H, Luis J Rutin inhibits proliferation, attenuates superoxide production and decreases adhesion and migration of human cancerous cells. Biomed Pharmacother 2016; 84: 1972–1978. 27829548 10.1016/j.biopha.2016.11.001

[19] Makino T, Kanemaru M, Okuyama S, Shimizu R, Tanaka H, Mizukami H Anti-allergic effects of enzymatically modified isoquercitrin (α-oligoglucosyl quercetin 3-*O*-glucoside), quercetin 3-*O*-glucoside, α-oligoglucosyl rutin, and quercetin, when administered orally to mice. J Nat Med 2013; 67: 881–886. 23494818 10.1007/s11418-013-0760-5

[20] Yeh SL, Lin YC, Lin YL, Li CC, Chuang CH Comparing the metabolism of quercetin in rats, mice and gerbils. Eur J Nutr 2016; 55: 413–422. 25691233 10.1007/s00394-015-0862-9

[21] Roza N, Possignolo L, Palanch A, Gontijo JA Effect of long-term high-fat diet intake on peripheral insulin sensibility, blood pressure, and renal function in female rats. Food Nutr Res 2016; 60: 28536. 26880072 10.3402/fnr.v60.28536 PMC4754019

[22] Nishiumi S, Ashida H Rapid preparation of a plasma membrane fraction from adipocytes and muscle cells: application to detection of translocated glucose transporter 4 on the plasma membrane. Biosci Biotechnol Biochem 2007; 71: 2343–2346. 17827673 10.1271/bbb.70342

[23] Zhang T, Yamashita Y, Yasuda M, Yamamoto N, Ashida H Ashitaba (*Angelica keiskei*) extract prevents adiposity in high-fat diet-fed C57BL/6 mice. Food Funct 2015; 6: 135–145. 25406632 10.1039/c4fo00525b

[24] Jiang H Yoshioka Y Yuan S Enzymatically modified isoquercitrin promotes energy metabolism through activating AMPKα in male C57BL/6 mice. Food Funct 2019; 10: 5188–5202. 31380532 10.1039/c9fo01008d

[25] Pang J, Choi Y, Park T Ilex paraguariensis extract ameliorates obesity induced by high-fat diet: potential role of AMPK in the visceral adipose tissue. Arch Biochem Biophys 2008; 476: 178–185. 18314006 10.1016/j.abb.2008.02.019

[26] Nishiumi S Bessyo H Kubo M Green and black tea suppress hyperglycemia and insulin resistance by retaining the expression of glucose transporter 4 in muscle of high-fat diet-fed C57BL/6J mice. J Agric Food Chem 2010; 58: 12916–12923. 21105694 10.1021/jf102840w

[27] Jiang H, Yamashita Y, Nakamura A, Croft K, Ashida H Quercetin and its metabolite isorhamnetin promote glucose uptake through different signaling pathways in myotubes. Sci Rep 2019; 9: 2690. 30804434 10.1038/s41598-019-38711-7 PMC6389993

[28] Puigserver P, Spiegelman BM Peroxisome proliferator-activated receptor-γ coactivator 1α (PGC-1α): transcriptional coactivator and metabolic regulator. Endocr Rev 2003; 24: 78–90. 12588810 10.1210/er.2002-0012

[29] Chi J, Cohen P The multifaceted roles of PRDM16: adipose biology and beyond. Trends Endocrinol Metab 2016; 27: 11–23. 26688472 10.1016/j.tem.2015.11.005

[30] Son MJ Kim WK Park A Set7/9, a methyltransferase, regulates the thermogenic program during brown adipocyte differentiation through the modulation of p53 acetylation. Mol Cell Endocrinol 2016; 431: 46–53. 27132805 10.1016/j.mce.2016.04.022

[31] Mukai R Handa O Naito Y High-fat diet causes constipation in mice via decreasing colonic mucus. Dig Dis Sci 2019; 14. DOI: 10.1007/s10620-019-05954-3. 10.1007/s10620-019-05954-3 31728788

[32] de Boer VC Dihal AA van der Woude H Tissue distribution of quercetin in rats and pigs. J Nutr 2005; 135: 1718–1725. 15987855 10.1093/jn/135.7.1718

[33] O'Leary KA, Day AJ, Needs PW, Mellon FA, O'Brien NM, Williamson G Metabolism of quercetin-7-and quercetine-3-glucuronides by an *in vitro* hepatic model: the role of human β-glucuronidase, sulfotransferase, catechol-*O*-methyltransferase and multiresistant protein 2 (MRP2) in flavonoid metabolism. Biochem Pharmacol 2003; 65: 479–491. 12527341 10.1016/s0006-2952(02)01510-1

[34] Stewart LK, Wang Z, Ribnicky D, Soileau JL, Cefalu WT, Gettys TW Failure of dietary quercetin to alter the temporal progression of insulin resistance among tissues of C57BL/6J mice during the development of diet-induced obesity. Diabetologia 2009; 52: 514–523. 19142628 10.1007/s00125-008-1252-0 PMC2758024

[35] Jayachandran M, Zhang T, Ganesan K, Xu B, Chung SSM Isoquercitrin ameliorates hyperglycemia and regulates key enzymes of glucose metabolism via insulin signaling pathway in streptozotocin-induced diabetic rats. Eur J Pharmacol 2018; 829: 112–120. 29665363 10.1016/j.ejphar.2018.04.015

[36] Ahmed OM, Moneim AA, Yazid IA, Mahmoud AM Antihyperglycemic, antihyperlipidemic and antioxidant effects and the probable mechanisms of action of *Ruta graveolens* infusion and rutin in nicotinamide-streptozotocin-induced diabetic rats. Diabetol Croat 2010; 39: 15–35.

[37] Chen ZP, McConell GK, Michell BJ, Snow RJ, Canny BJ, Kemp BE AMPK signaling in contracting human skeletal muscle: acetyl-CoA carboxylase and NO synthase phosphorylation. Am J Physiol Endocrinol Metab 2000; 279: E1202–E1206. 11052978 10.1152/ajpendo.2000.279.5.E1202

[38] Neumann D Is TAK1 a direct upstream kinase of AMPK? Int J Mol Sci 2018; 19. pii: E2412. 30111748 10.3390/ijms19082412 PMC6121279

[39] Frayn KN, Arner P, Yki-Järvinen H Fatty acid metabolism in adipose tissue, muscle and liver in health and disease. Essays Biochem 2006; 42: 89–103. 17144882 10.1042/bse0420089

[40] Miyazaki T, Honda A, Ikegami T, Iida T, Matsuzaki Y Human-specific dual regulations of FXR-activation for reduction of fatty liver using *in vitro* cell culture model. J Clin Biochem Nutr 2019; 64: 112–123. 30936623 10.3164/jcbn.18-80 PMC6436045

[41] Cantó C, Auwerx J PGC-1alpha, SIRT1 and AMPK, an energy sensing network that controls energy expenditure. Curr Opin Lipidol 2009; 20: 98–105. 19276888 10.1097/MOL.0b013e328328d0a4 PMC3627054

[42] López M Lage R Saha AK Hypothalamic fatty acid metabolism mediates the orexigenic action of ghrelin. Cell Metab 2008; 7: 389–399. 18460330 10.1016/j.cmet.2008.03.006

[43] McGarry JD, Leatherman GF, Foster DW Carnitine palmitoyltransferase I. The site of inhibition of hepatic fatty acid oxidation by malonyl-CoA. J Biol Chem 1978; 253: 4128–4136. 659409

[44] Xu X, So JS, Park JG, Lee AH Transcriptional control of hepatic lipid metabolism by SREBP and ChREBP. Semin Liver Dis 2013; 33: 301–311. 24222088 10.1055/s-0033-1358523 PMC4035704

[45] Ye JM Iglesias MA Watson DG PPARα/γ ragaglitazar eliminates fatty liver and enhances insulin action in fat-fed rats in the absence of hepatomegaly. Am J Physiol Endocrinol Metab 2003; 284: E531–E540. 12556350 10.1152/ajpendo.00299.2002

[46] Darlington GJ, Ross SE, MacDougald OA The role of C/EBP genes in adipocyte differentiation. J Biol Chem 1998; 273: 30057–30060. 9804754 10.1074/jbc.273.46.30057

[47] Hwang CS, Mandrup S, MacDougald OA, Geiman DE, Lane MD Transcriptional activation of the mouse obese (ob) gene by CCAAT/enhancer binding protein alpha. Proc Natl Acad Sci U S A 1996; 93: 873–877. 8570651 10.1073/pnas.93.2.873 PMC40150

[48] Wu Z, Xie Y, Bucher NLR, Farmer SR Conditional ectopic expression of C/EBPβ in NIH-3T3 cells induces PPARγ and stimulates adipogenesis. Genes Dev 1995; 9: 2350–2363. 7557387 10.1101/gad.9.19.2350

[49] Ceseña TI, Cardinaux JR, Kwok R, Schwartz J CCAAT/enhancer-binding protein C/EBP) beta is acetylated at multiple lysines: acetylation of C/EBPbeta at lysine 39 modulates its ability to activate transcription. J Biol Chem 2007; 282: 956–967. 17110376 10.1074/jbc.M511451200

[50] Liu Y Zhang YD Guo L Protein inhibitor of activated STAT 1 (PIAS1) is identified as the SUMO E3 ligase of CCAAT/enhancer-binding protein β (C/EBPβ) during adipogenesis. Mol Cell Biol 2013; 33: 4606–4617. 24061474 10.1128/MCB.00723-13 PMC3838193

[51] Tang QQ Grønborg M Huang H Sequential phosphorylation of CCAAT enhancer-binding protein β by MAPK and glycogen synthase kinase 3β is required for adipogenesis. Proc Natl Acad Sci U S A 2005; 102: 9766–9771. 15985551 10.1073/pnas.0503891102 PMC1175002

[52] Mitani T, Watanabe S, Yoshioka Y, Katayama S, Nakamura S, Ashida H Theobromine suppresses adipogenesis through enhancement of CCAAT-enhancer-binding protein β degradation by adenosine receptor A1. Biochim Biophys Acta Mol Cell Res 2017; 1864: 2438–2448. 28965824 10.1016/j.bbamcr.2017.09.017

[53] Lee YJ, Bernstock JD, Nagaraja N, Ko B, Hallenbeck JM Global SUMOylation facilitates the multimodal neuroprotection afforded by quercetin against the deleterious effects of oxygen/glucose deprivation and the restoration of oxygen/glucose. J Neurochem 2016; 138: 101–116. 27087120 10.1111/jnc.13643 PMC4916017

[54] Lee YH, Mottillo EP, Granneman JG Adipose tissue plasticity from WAT to BAT and in between. Biochim Biophys Acta 2014; 1842: 358–369. 23688783 10.1016/j.bbadis.2013.05.011 PMC4435780

[55] Villarroya F, Cereijo R, Gavaldà-Navarro A, Villarroya J, Giralt M Inflammation of brown/beige adipose tissues in obesity and metabolic disease. J Intern Med 2018; 284: 492–504. 29923291 10.1111/joim.12803

[56] Choi H, Kim CS, Yu R Quercetin upregulates uncoupling protein 1 in white/brown adipose tissues through sympathetic stimulation. J Obes Metab Syndr 2018; 27: 102–109. 31089549 10.7570/jomes.2018.27.2.102 PMC6489452

[57] Rivera L, Morón R, Sánchez M, Zarzuelo A, Galisteo M Quercetin ameliorates metabolic syndrome and improves the inflammatory status in obese Zucker rats. Obesity (Silver Spring) 2008; 16: 2081–2087. 18551111 10.1038/oby.2008.315

[58] Kobori M, Masumote S, Akimoto Y, Oike H Chronic dietary intake of quercetin alleviates hepatic fat accumulation associated with consumption of a Western-style diet in C57/BL6J mice. Mol Nutr Food Res 2011; 55: 530–540. 21462320 10.1002/mnfr.201000392

[59] Gómez-Hernández A, Beneit N, Díaz-Castroverde S, Escribano Ó Differential role of adipose tissues in obesity and related metabolic and vascular complications. Int J Endocrinal 2016; 2016: 1216783. 10.1155/2016/1216783 PMC5059561 27766104

[60] Cunha CA Lira FS Rosa Neto JC Green tea extract supplementation induces the lipolytic pathway, attenuates obesity, and reduces low-grade inflammation in mice fed a high-fat diet. Mediators Inflamm 2013; 2013: 635470. 23431242 10.1155/2013/635470 PMC3569937

[61] Zuo Z, Zhang L, Zhou L, Chang Q, Chow M Intestinal absorption of hawthorn flavonoids—*in vitro*, *in situ* and *in vivo* correlations. Life Sci 2006; 79: 2455–2462. 16989871 10.1016/j.lfs.2006.08.014

